# Supplementary material for: HT-eQTL: integrative expression quantitative trait loci analysis in a large number of human tissues
Source: BMC Bioinformatics. 2018 Mar 9;19:95. doi: 10.1186/s12859-018-2088-3 (PMC5845327; doi:10.1186/s12859-018-2088-3)
Supplement: Supplementary file 1 — Supplementary material of the HT-eQTL model fitting procedure. (PDF 144 kb) [file 12859_2018_2088_MOESM1_ESM.pdf]

HT-eQTL: Integrative Expression Quantitative Trait Loci Analysis in a Large Number of Human Tissues: Supplementary Materials

A HT-eQTL Model Fitting Procedure

In this section, we provide more details about the HT-eQTL model fitting procedure. We first elaborate a modified EM algorithm for the parameter estimation of a two-tissue MT-eQTL model. Then we provide the flowchart of the proposed HT-eQTL method.

A.1 Modified EM Algorithm for Two-Tissue Model

Let $\mathbf{Z}_\lambda \in \mathbb{R}^2$ be a vector of z-scores in two tissues for the gene-SNP pair indexed by $\lambda \in \Lambda$. We assume the z-score vectors for different gene-SNP pairs are independent. The joint log likelihood of the observed data $\{\mathbf{z}_\lambda\}$ is

$$L(\theta) = \sum_{\lambda \in \Lambda} \log \left(\sum_{\gamma \in \{0,1\}^2} p(\gamma) f_\gamma(\mathbf{z}_\lambda, \theta) \right), \tag{S.1}$$

where $\theta = \{\mathbf{p}, \boldsymbol{\mu}, \boldsymbol{\Delta}, \boldsymbol{\Sigma}\}$ is the collection of model parameters, and $f_\gamma(\cdot)$ is the probability density function (pdf) of the bivariate Gaussian distribution $\mathcal{N}(\boldsymbol{\mu} \cdot \gamma, \boldsymbol{\Delta} + \boldsymbol{\Sigma} \cdot \gamma\gamma')$. We shall introduce a modified EM algorithm to maximize (S.1) with respect to θ. (In fact, all the model parameters in θ only concern the marginal distribution of $\mathbf{Z}_\lambda$. Even without the independence assumption between different gene-SNP pairs, the log likelihood function (S.1) can be viewed as a log-transformed marginal composite likelihood, and still can be used to estimate the model parameters. For simplicity, we continue our discussion under the independence assumption.)

In order to maximize (S.1), we exploit an EM algorithm. We treat the underlying eQTL configuration vector $\boldsymbol{\Gamma}_\lambda$ as the latent variable. It may only

take four different values $(0,0), (0,1), (1,0), (1,1)$, and the prior probabilities are contained in $\mathbf{p}$. The posterior distribution of $\mathbf{\Gamma}_\lambda$ given $\mathbf{Z}_\lambda$ is

$$\mathbb{P}(\mathbf{\Gamma}_\lambda = \gamma_0 | \mathbf{Z}_\lambda = \mathbf{z}_\lambda) = \frac{p(\gamma_0) f_{\gamma_0}(\mathbf{z}_\lambda, \theta)}{\sum_{\gamma \in \{0,1\}^2} p(\gamma) f_\gamma(\mathbf{z}_\lambda, \theta)}. \tag{S.2}$$

In the E step, we evaluate the above posterior probabilities under the current estimate of the parameters; in the M step, we optimize the conditional expectation of the joint log likelihood of $\mathbf{Z}_\lambda$ and $\mathbf{\Gamma}_\lambda$.

More specifically, the estimation of $\mathbf{p}$ has a closed-form solution in the M step

$$\widehat{p(\gamma)} = \sum_{\lambda \in \Lambda} p(\gamma | \mathbf{z}_\lambda, \theta^{(t)}) / |\Lambda|, \ \gamma \in \{0,1\}^2 \tag{S.3}$$

where $p(\gamma | \mathbf{z}_\lambda, \theta^{(t)})$ is the posterior probability with respect to the configuration γ calculated from the previous estimate $\theta^{(t)}$, and $|\Lambda|$ is the cardinality of Λ. However, $\boldsymbol{\mu}, \mathbf{\Delta}, \mathbf{\Sigma}$ do not have closed-form estimates. We notice

$$\mathbf{Z}_\lambda | (\mathbf{\Gamma}_\lambda = (0,0)) \sim \mathcal{N}(\mathbf{0}, \mathbf{\Delta}), \ \mathbf{Z}_\lambda | (\mathbf{\Gamma}_\lambda = (1,1)) \sim \mathcal{N}(\boldsymbol{\mu}, \mathbf{\Delta} + \mathbf{\Sigma}).$$

Namely, the model parameters $\boldsymbol{\mu}, \mathbf{\Delta}, \mathbf{\Sigma}$ are readily separable under two configurations $\mathbf{0} = (0,0)$ and $\mathbf{1} = (1,1)$. Moreover, if we only focus on the two configurations in the objective function, we can obtain closed-form estimates of all the parameters as

$$\begin{aligned}
\widehat{\mathbf{\Delta}} &= \sum_{\lambda \in \Lambda} p(\mathbf{0} | \mathbf{z}_\lambda, \theta^{(t)}) \mathbf{z}_\lambda \mathbf{z}_\lambda' / \sum_{\lambda \in \Lambda} p(\mathbf{0} | \mathbf{z}_\lambda, \theta^{(t)}), && \text{(S.4)} \\
\widehat{\boldsymbol{\mu}} &= \sum_{\lambda \in \Lambda} p(\mathbf{1} | \mathbf{z}_\lambda, \theta^{(t)}) \mathbf{z}_\lambda / \sum_{\lambda \in \Lambda} p(\mathbf{1} | \mathbf{z}_\lambda, \theta^{(t)}), && \text{(S.5)} \\
\widehat{\mathbf{\Delta}} + \widehat{\mathbf{\Sigma}} &= \sum_{\lambda \in \Lambda} p(\mathbf{1} | \mathbf{z}_\lambda, \theta^{(t)}) (\mathbf{z}_\lambda - \widehat{\boldsymbol{\mu}})(\mathbf{z}_\lambda - \widehat{\boldsymbol{\mu}})' / \sum_{\lambda \in \Lambda} p(\mathbf{1} | \mathbf{z}_\lambda, \theta^{(t)}). && \text{(S.6)}
\end{aligned}$$

In practice, the two configurations $\mathbf{0}$ and $\mathbf{1}$ always have dominant probabilities. Therefore, we do not lose much accuracy by restricting our focus on the two terms in the estimation of $\boldsymbol{\mu}, \mathbf{\Delta}, \mathbf{\Sigma}$. The modified M step has closed form solutions, and thus the computation is highly efficient.

To obtain the final estimate of θ, we iterate between the E step and the M step until convergence.

A.2 Flowchart of HT-eQTL

The HT-eQTL approach consists of two steps: the fitting of two-tissue MT-eQTL models for all pairs of tissues; the assembling of model parameters. The first step has been described in the previous section, and the second step was discussed in the main paper. Here we provide the flowchart of entire procedure.

Algorithm S.1 Flowchart of HT-eQTL Model Fitting

Input K-dimensional z-scores for gene-SNP pairs in Λ;
Step I: Fit two-tissue models
while The log likelihood difference has not reached convergence **do**

- E step: Evaluate the posterior probabilities in (S.2)
- M step: Obtain the estimates in (S.3)–(S.6)

end while
Step II: Assemble parameters

- Estimate $\mathbf{\Delta}$ by collecting individual correlations
- Estimate $\mathbf{\Sigma}$
  - Estimate the diagonal values by taking the minimum of all candidates
  - Estimate the underlying correlation matrix by collecting individual correlations
- Estimate $\mathbf{p}$ (multi-Probit model)
  - Derive a bivariate Gaussian distribution from each two-tissue model
  - Assemble a K-variate Gaussian distribution
  - Evaluate different cumulative probabilities of the derived distribution
  - Threshold the individual components of $\mathbf{p}$ and renormalize
- Estimate $\boldsymbol{\mu}$ by taking the average of all candidates

---
